# Evaluating the effectiveness of a schools-based programme to promote exercise self-efficacy in children and young people with risk factors for obesity: Steps to active kids (STAK)

**DOI:** 10.1186/1471-2458-11-830

**Published:** 2011-10-26

**Authors:** Cris Glazebrook, Martin J Batty, Nivette Mullan, Ian MacDonald, Dilip Nathan, Kapil Sayal, Alan Smyth, Min Yang, Boliang Guo, Chris Hollis

**Affiliations:** 1 School of Community Health Sciences, Division of Psychiatry, University of Nottingham, Derby Road, Nottingham, NG7 2UH, UK; 2 School of Biomedical Sciences, University of Nottingham, Derby Road, Nottingham, NG7 2UH, UK; 3 Community Paediatrics, Nottinghamshire University NHS Hospitals Trust Queen's Medical Centre, Derby Road, Nottingham, NG7 2UH, UK; 4 Division of Respiratory Medicine, Nottingham City Hospital, Hucknall Road, Nottingham, NG5 1PB, UK; 5 School of Community Health Sciences, Division of Psychiatry, University of Nottingham, 9 Triumph Road, Nottingham, NG7 2GT, UK

## Abstract

**Background:**

Low levels of physical activity in children have been linked to an increased risk of obesity, but many children lack confidence in relation to exercise (exercise self-efficacy). Factors which can impact on confidence include a chronic health condition such as asthma, poor motor skills and being overweight. Increasing levels of physical activity have obvious benefits for children with asthma and children who are overweight, but few activity interventions with children specifically target children with low exercise self-efficacy (ESE). This study aims to evaluate the efficacy and feasibility of a schools-based activity programme suitable for children with risk factors for adult obesity, including asthma, overweight and low exercise self-efficacy.

**Methods/Design:**

A clustered (at the level of school) RCT will be used to compare a targeted, 10 week, stepped activity programme (activity diary, dance DVD, circuit-training and motivational interviewing) designed to promote ESE. We will recruit 20 primary schools to participate in the intervention and 9-11 year old children will be screened for low levels of ESE, asthma and overweight. In order to provide sufficient power to detect a difference in primary outcomes (Body Mass Index-BMI & ESE at 12 month follow-up) between children in the intervention schools and control schools, the target sample size is 396. Assessments of BMI, ESE, waist circumference, peak flow, activity levels and emotional and behavioural difficulties will be made at baseline, 4 months and 12 month follow-up.

**Discussion:**

We aim to increase ESE and levels of physical activity in children with risk factors for adult obesity. The outcomes of this study will inform policy makers about the feasibility, acceptability and effectiveness of delivering targeted health interventions within a school setting.

**Trial Registration:**

ISRCTN Register no. ISRCTN12650001

## Background

The increasing prevalence of children and young people classified as overweight or obese presents a significant challenge to public health authorities. Obesity is often chronic and is associated with increased morbidity, including heart disease and diabetes [1]. Around 10% of the world's school children are estimated to be overweight, with higher rates still in countries with well-developed economies [2]. The national child measurement programme in England (2009/10) found that a third of children in year 6 (age 10 to 11 years) are currently overweight or obese. The risk of obesity increases through childhood with 19% of 10 to 11 year olds above the 95^th ^centile of the 1990 weight charts, compared to 10% in reception classes. The global and national trend of increasing rates of obesity is mirrored here in the East Midlands. Children aged 10-11 years have higher levels of obesity compared to the national average, particularly in girls [3]. Health status is a key factor in determining exercise behaviour. In the East Midlands, only 9% of those with a limiting, longstanding illness reported taking regular exercise compared to 23% of those without longstanding health problems [4]. Other evidence suggests that chronic health conditions act as a barrier to exercise and are a risk factor for obesity in children and young people [5].

The UK has one of the highest rates of childhood asthma in Europe and the ISAAC study [6] found nearly 1 in 5 12-14 year olds reported treatment for asthma in the previous year. Poorly controlled asthma in childhood is associated with significant psychological and social burden. Data from a national survey of mental health in 10,438 children aged 5-15 years showed that children with asthma and in poor health were 3.5 times more likely to receive a diagnosis of depression or anxiety than children without health problems [7]. The relationship between asthma and mental health may be mediated by the impact of the condition on schooling and recent research has found that children with asthma are more likely to have missed school in the previous year than their peers [8]. Children with asthma were found to have specific social difficulties and were more likely to report not having a group of friends to socialise with and unhappiness at school. Increasing physical activity may have particular benefits for children with health problems such as asthma, since increased levels of physical activity are associated with better mental and physical health [9]. Furthermore, children with asthma who were more active were found to have fewer emotional and behavioural difficulties [10].

Children attending paediatric clinics for the treatment of asthma were found to be less active than children attending other paediatric clinics, with both parents and children typically perceiving asthma as a significant barrier to activity. The same study found that children with asthma were also more likely to be overweight, with 21% being classified as obese [10]. This represents a significant co-morbidity, as overweight children are less likely to experience remission of asthma symptoms in adolescence [11]. In obese adults with asthma, weight is strongly associated with severity of symptoms, and a large US study of children aged 4 to 9 years with asthma found that obese children had more emergency room visits than their normal weight counterparts [12]. Even modest weight reductions are associated with improved lung function [13]. Increasing physical activity can also have direct benefits for children with asthma by improving lung function and reducing the symptoms of exercise induced wheeze [14].

One mechanism by which asthma and other chronic health conditions such as obesity reduce activity levels is through their impact on children's confidence in relation to exercise, or exercise self-efficacy (ESE). For example, children who performed poorest on a shuttle run task were those who were both overweight and had low exercise self-efficacy. Exercise self-efficacy (ESE) has been shown to increase following successful completion of a physical exercise challenge [15], suggesting that exercise self-efficacy may improve following positive experiences of physical activity. This is important since exercise self-efficacy is strongly predictive of future levels of physical activity [16]. Exercise self-efficacy is also a predictor of perceptions of exercise, independent of the actual nature of the exercise itself. For example, in a sample of girls aged 8 to 15 years, those with lower exercise self-efficacy rated a cycling task as harder regardless of their actual energy expenditure [15]. The aim of present study is to evaluate whether a targeted intervention to promote exercise self-efficacy in children with risk factors for adult obesity can be successfully implemented in primary schools and can improve ESE, BMI, mental health and lung function in children.

## Objective

To evaluate the efficacy and feasibility of a schools-based activity programme suitable for children with risk factors for adult obesity, including asthma, overweight and low exercise self-efficacy.

It is hypothesised that children receiving the intervention will have increased exercise self-efficacy, lower BMI, lower waist circumference, increased levels of physical activity and improved lung function at follow-up compared to children in the control group.

## Methods/Design

The study will be a cluster randomised controlled trial (RCT). Primary schools (*n *= 20) in Nottinghamshire and Derbyshire will be randomised to intervention or control, clustered at the level of the school. Schools will be pair-wise matched for demographic characteristics (number of pupils, ethnic profile and denomination (if faith schools), indices of social deprivation http://www.neighbourhood.statistics.gov.uk and SAT results http://www.education.gov.uk/performancetables/primary_10.shtml). Schools in each pair will be randomised to intervention or control by the clinical trials unit at The University of Nottingham following collection of baseline data.

### Sample and Inclusion/exclusion criteria

Participants will be children aged 9 to 11 years with risk factors for adult obesity including asthma, overweight or low levels of exercise self-efficacy. Participants will be recruited and screened in local schools for eligibility for the study.

Inclusion criteria will be children who meet one or more of the following: child-rated asthma, child-rated low exercise self-efficacy, teacher-rated build at or above the 75^th ^centile on the 1990 UK BMI centiles [17], or teacher-rated concern about the child's difficulties with participation in P.E classes. Exclusion criteria will be high levels of customary activity as indicated by teacher or child report.

### Screening

Primary schools in Nottinghamshire and Derbyshire will be invited to take part in the intervention. Parents/carers with children in the target age range will be sent letters explaining the study and the screening process for inclusion. Parents will be given the opportunity to opt out of the study at this point. Children whose parents have not opted out will be screened in school. Teachers will rate each child's build using the Child Body Image Scale (CBIS) [18]. They will also rate each child's participation in physical education classes, including whether the child could be considered "sporty", any difficulties they may have associated with health conditions, lack of confidence, lack of coordination or lack of concentration. The child's screening questionnaire will probe whether they have asthma, whether they receive treatment for asthma and whether they participate in any sports teams. Children will also rate their exercise self-efficacy using the predilection sub-scale of the CSSAPA [16]. Parents of children who meet the inclusion and exclusion criteria will be sent further information about the study, a consent form and information about the study for the child.

### Measures

#### Demographic characteristics

Questions about parents' occupation and educational level. Child's ethnic origin and family composition.

#### Asthma health questionnaire

Questions on asthma treatment, visits to GP for asthma, days missed from school for asthma, hospital visits for asthma.

#### Height

A stadiometer will be used to measure height in cms, with the child standing upright with bare feet and their back in contact with the stadiometer.

#### Weight measurement

The child will be weighed with minimal clothing and their shoes removed. The scale will be checked to ensure that it is reading zero and the child will be asked to stand on the centre of the scales, without support and with their weight distributed evenly on both feet. Weight will be record in kgs. BMI will also be calculated (weight/height^2^).

#### Waist circumference

The tape will be positioned equidistant between the iliac crest and the base of the rib cage and the waist circumference recorded in cms.

#### Lung function

Peak expiratory flow will be measured by peak flow meter (best of three attempts) and percentage of predicted peak expiratory flow rate for height, age and gender will also be calculated.

#### Physical Activity Questionnaire (PAQ) [19]

The PAQ consists of 55 items and will be used to assess the frequency of different types of physical activity. The questionnaire was initially developed for a study aimed at lowering obesity in American-Indian children and modified for use in the UK, where it was found to discriminate activity levels between children attending asthma clinics and children attending other paediatric out-patient clinics [10]. Children will be asked to rate a range of activities, both active and sedentary, on a three point scale (none, a little, a lot) at three time points in the previous 24 hours (today before school, yesterday after school, yesterday during school). Scores will be summed to form a total score for sedentary activities (possible range 14-42) and a total score for physical activities (possible range 41-123), with higher scores indicating greater activity.

#### Strengths and Difficulties Questionnaire (SDQ) - Parent & Teacher versions [20]

The SDQ is used to assess emotional and behavioural difficulties in children aged 4- 16 years. The scale consists of 25 items, divided into five subscales (conduct problems, hyperactivity/inattention, emotional symptoms, peer problems and pro-social behaviours), each of which contain 5 items and are rated on a 3-point scale. Conduct problems, hyperactivity, emotional symptoms and peer problem scores are summed to generate a total difficulties score. Teachers and parents will be asked to complete the SDQ and the one page impact supplement to establish the impact of any emotional and behavioural symptoms on day to day life.

#### Children's Self-Perceptions of Adequacy in and Predilection for Physical Activity scale CSAPPA [16]

The CSAPPA scale comprises 19 items to measure children's self--perception of their adequacy and ability in performing exercise and their desire to join physical activities in general. It was designed for children aged between 9 -16 years. Scores for total self-efficacy range from 19 to 76, with higher scores indicating better exercise self-efficacy. Total scores below 60 indicate a level below that expected for moderate self-efficacy. The scale comprises three sub-scales: adequacy (confidence in physical activity), predilection (preference for physical activity over sedentary activities) and enjoyment of physical education class. The CSAPPA has been found to have reasonable construct validity and excellent test re-test reliability [16]. Furthermore, the scale's predictive validity is high and the scales have good internal consistency [21,22].

### Procedure

Parents of all children will complete the demographic questionnaire at baseline. Parents will be sent the SDQ and the asthma health questionnaire at baseline, 4 month and 12 month follow-up. Teachers will also be asked to complete the SDQ at baseline and 4 and 12 month follow-up. Children's physiological measures (weight, height, waist circumference and peak flow) and questionnaire measures (PAQ and ES) will be completed in school at baseline and at 4 and 12 months follow-up.

### Steps to Active Kids (STAK) activity programme

A stepped approach will be used for the intervention. All children in the intervention group will complete Steps 1 and 2. Children with high levels of risk factors will be invited to take part in Step 3.

#### Step 1

Activity diary and dance DVD. Children will be given a loose leaf exercise diary with colour coded informational inserts (e.g. exercise with asthma) and inserts to record daily activities. The diary has been designed in consultation with children from a pilot school to be engaging and easy to use. Children will be encouraged to personalise their folder and a number of fun interactive pages will also be included. Using the well-publicised UK target of 'five a day' for eating fruit and vegetables, we aim for each child to complete five 'pieces' of physical activity a day, thereby meeting the Department of Health target of a minimum of 60 minutes physical activity per day for children [23] (e.g. walking quickly to school, 10 minutes skipping, running at playtime). Children collect 1 point for each activity and can record these points in their daily activity log. The activity diary is reviewed each week in class and children's efforts to increase their levels of physical activity are reinforced with stickers and verbal encouragement.

To complement the diary we have developed a 'Street Dance' DVD specifically for the programme. This comprises 10 minute daily exercise sessions, including a warm up, learning a new dance move and a cool down to cover a one week period. Each 10 minute segment earns 1 activity point. Sessions have been designed to be short, simple and intensive and have been modelled by boys and girls from an ethnically diverse group of children with a range of body sizes. The 28 short dance routines build up slowly to a complete dance. An information insert accompanies each DVD and a letter to parents encourages them to support their children learning the dance.

#### Step 2

The group classes for Step 2 will be held during the school day and will incorporate a circuit training approach, with a sequence of 12 two-minute activities such as throwing and catching a ball, skipping, stepping on and off a box, swing ball, bouncing on a trampette and wii fit^©^. Each session will last about 50 minutes and include an activity diary check. The circuit training was designed by a paediatric physiotherapist and will be run by a sports coach once a week for 4 weeks. Trained volunteers will assist the sports coach in encouraging the children and helping them to record their achievements for the different stations (e.g. number of skips, metres rowed etc).

#### Step 3

Children who are above the 91^st ^centile at baseline will move on to Step 3. Participants in Step 3 will complete two ten-minute sessions. Each session will comprise an individual meeting with a researcher trained in motivational interview techniques. In the first session, agreed goals will be set, which will be reviewed 7 days later during the second session. Progress of these goals will be reviewed during the 4 month follow up.

The theoretical basis for the intervention is Bandura's social learning theory [24], which argues that behaviour change is facilitated by observing successful role models. To be effective the child must identify with the role model(s). This is achieved in the intervention by targeting the intervention only to children who have similar barriers to exercise. Similarly the dance routines on the DVD are modelled by children from a local primary school, with no previous experience of dance, successfully learning the routines. Bandura proposes that self-efficacy is also key to behaviour change and that this can be facilitated by providing "mastery" experiences. In the context of STAK, this involves helping the children to derive a sense of competence associated with physical activity. The STAK intervention promotes mastery through its simple DVD, clear and attainable activity targets and circuit training to promote skills development and a sense of accomplishment. As social learning theory also suggests that behaviour can be changed by persuasion, the intervention involves appropriately formatted information and motivational interviews.

Children in the control group will not take part in the intervention, but control schools will be given the materials for Step 1 at the end of the study.

### Outcome Measures

Primary outcome measures will be body mass index and exercise self-efficacy at 12 month follow-up.

Secondary outcomes will be self-reported activity, emotional and behavioural well-being, lung function and waist circumference at 4 and 12 month follow-up.

### Sample size and Justification

A preliminary study showed the intra class correlation for the self-efficiency measure among schools was 6.5%. In the cluster randomised design with an average of 20 students available in each school, to detect a half standard deviation difference in means of 4 points (i.e. between 49.6 and 53.6 with SD = 8.1) at the 5% level of significance with 80% power, approximately 17 schools and 340 students in total would be required [25]. The intra class correlation for BMI measure is 0.6%, therefore to detect the half standard deviation difference of 2.3 BMI change at 5% level of significance with 80% power would require 8 schools and 160 students in total. Assuming an overall 10% drop-out rate of students and potential school withdraw, for self-efficiency measure, a total of 396 students with a mean of 22 per school for 18 schools would be required in this study.

### Randomisation and Blinding

All baseline assessments will be undertaken prior to randomisation. Upon completion of baseline assessments, the names of the schools will be forwarded to the Programme Manager for entry into a randomisation programme developed by the Clinical Trials Unit at The University of Nottingham. Schools will be allocated to either condition (intervention or control) with equal probability. At the end of the study, children in the control schools will be offered the educational materials.

### Statistical Analysis

The outcome data present a 3-level hierarchical structure due to the cluster randomisation and follow-up design, i.e. students (level 2 unit) nested within schools (level 3 unit) and repeated measures at 3 time points (level 1 unit) are nested within students. Treatment status is school level variable, together with some student level variables such as gender, age and other background variables. Multilevel modelling will be applied to examine the treatment effects on the primary outcome measures [26], taking into account variability of outcomes between school and between students. The student's weight category (overweight at baseline if =>91^st ^centile) and status of having asthma (yes/no) could also be included in the model as student level (level 2) exploratory variables. The intervention effects in the outcome measure differentiated by these group of students will be tested in the multilevel models analysis. Since both primary outcomes are continuous variable, multilevel linear models will be considered. The computational algorithm in multilevel modelling takes care of missing outcome automatically under missing at random (MAR) assumption. However, we shall examine missing value pattern by multilevel logistic regression to make sure missing data are not dependent on key variables such as deprivation or overweight. Sensitivity analyses will be performed if missing data are not missing at random.

Analyses for secondary outcome variables will be conducted in similar manner.

### Ethics

The study received ethical approval from The University of Nottingham Medical School Ethics Committee (B/10/2009) and is currently recruiting participants.

## Discussion

Results from the study will show whether a targeted activity intervention is effective and feasible. Outputs from the study will include a fully developed intervention with supporting materials.

## List of abbreviations

BMI: Body Mass Index; CBIS: Child Body Image Scale; CLAHRC: Collaborations for Leadership in Applied Health Research and Care; CSSAPA: Children's Self-Perceptions of Adequacy in and Predilection for Physical Activity scale; DoH: Department of Health; NDL: Nottinghamshire, Derbyshire, Lincolnshire; PAQ: Physical Activities Questionnaire; RCT: Randomised Control Trial; SDQ: Strengths and Difficulties Questionnaire.

## Competing interests

The authors declare that they have no competing interests.

## Authors' contributions

All authors contributed to the design of the study, the study protocol and the writing up of the paper. All authors read and approved the final manuscript.

CG wrote the project application for funding.

## Pre-publication history

The pre-publication history for this paper can be accessed here:

http://www.biomedcentral.com/1471-2458/11/830/prepub
